# Reliability of the Glasgow Antipsychotic Side-effects Scale for Clozapine Japanese version (GASS-C-J)

**DOI:** 10.1371/journal.pone.0234864

**Published:** 2020-06-18

**Authors:** Kohei Kitagawa, Ryuhei So, Nobuyuki Nomura, Yuya Mizuno, Fuminari Misawa, Masafumi Kodama, Hiroyuki Uchida, Masaru Mimura, Hiroyoshi Takeuchi

**Affiliations:** 1 Department of Neuropsychiatry, Okayama Psychiatric Medical Center, Okayama, Japan; 2 Department of Neuropsychiatry, Yamanashi Prefectural Kita Hospital, Yamanashi, Japan; 3 Department of Neuropsychiatry, Keio University School of Medicine, Tokyo, Japan; 4 Department of Psychosis Studies, Institute of Psychiatry, Psychology & Neuroscience, Kings College London, London, United Kingdom; Tokyo Metropolitan Institute of Medical Science, JAPAN

## Abstract

The purpose of this study was to develop the Glasgow Antipsychotic Side effects Scale for Clozapine Japanese version (GASS-C-J) and examine its reliability to assess clozapine-related side effects. We developed the GASS-C-J using forward and backward translation. Semantic equivalence of the GASS-C-J to the GASS-C was confirmed by the original author. We then administered the GASS-C-J twice to 109 patients on clozapine treatment at two psychiatric hospitals in Japan. We assessed the internal consistency and test-retest reliability of the GASS-C-J using Cronbach’s alpha and weighted kappa coefficient, respectively. We also examined if discrepancies in each GASS-C-J item score between the first and second rating were correlated with items of the Brief Evaluation of Psychosis Symptom Domains (BE-PSD). The Cronbach’s alpha coefficient of the GASS-C-J at the first and second rating was 0.78 (95% CI: 0.72 to 0.84) and 0.82 (95% CI: 0.76 to 0.88), respectively. The weighted kappa coefficient of individual and total GASS-C-J item scores ranged from 0.45 to 0.88. Some symptom domains were correlated with discrepancies in specific items of the GASS-C-J: psychotic symptoms and nausea/vomiting (r_s_ = 0.27), thirst (r_s_ = 0.31), and appetite/weight gain (r_s_ = 0.27); disorganized thinking and urinary incontinence (r_s_ = 0.26); depression/anxiety and myoclonus (r_s_ = 0.25), hypersalivation (r_s_ = -0.27), and blurred vision (r_s_ = -0.22). These findings demonstrate that the GASS-C-J can be used in clinical and research settings as a reliable scale to assess clozapine-related side effects.

## Introduction

Clozapine is shown to be superior to other antipsychotics for efficacy [1–5] and is the only licensed medication for treatment-resistant schizophrenia [6,7]. Given that discontinuing clozapine or switching from clozapine to another antipsychotic has been reported to worsen psychiatric symptoms [8–10], it is critically important to continue clozapine for treatment-resistant schizophrenia where possible.

However, patients frequently withdraw from treatment of clozapine for various reasons [11–15]. Among them, side effects are the most common reasons for discontinuation of clozapine [16] which suggests that early detection and management of clozapine-associated side effects is vital in enabling continued treatment. To this point, using a checklist to both systematically and efficiently assess side effects is expected to be a useful strategy.

The Glasgow Antipsychotic Side-effects Scale (GASS) [17] is used as a scale for evaluating common side effects of antipsychotic drugs. However, since clozapine has a unique side effect profile such as myoclonus, hypersalivation, and frequent urination, the GASS is considered insufficient to detect clozapine-related side effects comprehensively. To effectively screen for clozapine-associated side effects, the Glasgow Antipsychotic Side effects Scale for Clozapine (GASS-C) was developed based on the GASS [18]. The GASS-C is a simple but comprehensive scale that can be completed by patients in approximately five minutes, which can then be used in the clinic to discuss unpleasant side effects and guide clinical decision making. Having access to the GASS-C is expected to be particularly important in Japan where clozapine is extremely underutilized [19] and many psychiatrists still lack experience in managing clozapine-related side effects. To address this clinically relevant issue, in the present study, we aimed to develop the GASS-C Japanese version (GASS-C-J) and examine its reliability.

## Methods

### Study design and subjects

We assessed the reliability of the GASS-C-J using data from a cross-sectional study aiming to investigate the relationship between plasma concentrations of clozapine and its side effects in Japanese patients with treatment-resistant schizophrenia. The study was carried out in Yamanashi Prefectural Kita Hospital and Okayama Psychiatric Medical Center, Japan. The institutional review boards at both hospitals approved the study protocol.

Individuals who met the following criteria were included in the study: (1) aged 20 years or older; (2) diagnosed with schizophrenia according to International Statistical Classification of Diseases and Related Health Problems, 10th Revision (ICD-10) [20]; (3) being treatment-resistant defined as an insufficient response to at least two adequate antipsychotic trials (i.e., treatment with chlorpromazine equivalent doses of ≥600 mg/day for ≥4 weeks, and at least one trial using an atypical antipsychotic) or treatment-intolerant (i.e. not being able to tolerate atypical antipsychotics due to side effects); (4) receiving outpatient or inpatient treatment at either hospital between September 2018 and February 2019; and (5) being capable of participating in the study judged by their attending physician. After complete explanation regarding the study, the participants provided written informed consent.

### Study instruments

#### Development of GASS-C Japanese version (GASS-C-J)

The GASS-C is a self-rated questionnaire designed to elicit the subjectively unpleasant side effects of clozapine during the past week. The GASS-C is composed of 16 questions with the following four responses: “0 –Never”; “1 –Once”; “2 –A few times”; and “3 –Every day”. A total score of the GASS-C is calculated by adding all item scores, ranging from 0 to 48. According to the original GASS-C [18], the total GASS-C score is interpreted as follows: “0–16 = absent/mild side effects”; “17–32 = moderate side effects”; and “33–48 = severe side effects”.

Three authors (N.N., F.M., and H.T.) independently translated the original English version of the GASS-C into Japanese with permission from the original author, Dr. Hynes, and integrated the three Japanese versions into one. Any disagreement was resolved through discussion. Another author (Y.M.), a Japanese/English bilingual speaker, performed the backward translation. The semantic equivalence of the backward translated scale and the original GASS-C was confirmed by Dr. Hynes.

#### Brief evaluation of psychosis symptom domains (BE-PSD)

The BE-PSD is a clinician-rated rating scale, designed to capture the five symptom domains of psychosis: (1) psychotic symptoms; (2) disorganized thinking; (3) negative symptoms; (4) excitement/mania; and (5) depression/anxiety [21]. The severity of each symptom domain is scored based on the frequency of symptoms and their impact on behavior during the past week or interview, with scores ranging from 0 (absent) to 6 (very severe). The sum of the five symptom scores, ranging from 0 to 30, is used as a measure of overall symptom severity. In this study, we explored whether severity of specific symptom domains as assessed by the BE-PSD affects the test-retest reliability of the GASS-C-J.

### Study procedures

Subjects were asked to complete the GASS-C-J twice with an interval of 14 days or less. The BE-PSD was evaluated by the attending physician when the informed consent was obtained.

### Statistical analyses

The internal consistency of the GASS-C-J was evaluated using Cronbach’s alpha coefficient at the first and second test rating. The test-retest reliability of the total and individual item scores of the GASS-C-J were assessed using weighted kappa coefficient. Cases with missing data and those where prescriptions changed between the first and second rating were excluded from the analyses. Correlations between the discrepancies in individual GASS-C-J item scores between the first and second rating, and BE-PSD symptom domain scores were analyzed using Spearman’s correlation coefficient. All statistical analyses were conducted using R version 3.6.0 (R Core Team, 2017).

## Results

### Participants

A total of 151 patients met the eligibility criteria. Of these, 109 patients were included in the study and 42 patients refused to participate. Demographic and clinical characteristics of the included patients are shown in Table 1. The internal consistency of the GASS-C-J was examined using data from the all patients. The test-retest reliability of the GASS-C-J was examined in 99 patients. Ten patients were excluded from the analysis due to change in their clozapine dose between the two ratings.

**Table 1:** Demographic and clinical characteristics of patients (N = 109).

	Mean (SD) or N (%)	Reference range*
Okayama	60 (55.0)	
Yamanashi	49 (45.0)	
Inpatients	28 (25.7)	
Sex, male	61 (56.5)	
Age, years	43.0 (10.2)	
BMI	24.8 (4.7)	
Waist circumference (cm)	88.9 (11.2)	
Smokers	42 (39.0)	
Cigarettes per day per smokers	19.8 (13.9)	
Duration of clozapine treatment, years	4.1 (2.4)	
Dose of clozapine (mg/day)	357.0 (137.9)	
Those reporting constipation	60 (55.6)	
Those receiving laxatives	84 (82.4)	
White blood cell count (μL)	6936 (2080)	3300–8600
Non-fasting blood glucose (mg/dL)	112.3 (23.9)	< 200
GASS-C-J in the first rating		
0–16 = absent/mild side effects	65 (59.6)	
17–32 = moderate side effects	39 (35.8)	
33–48 = severe side effects	4 (3.7)	
BE-PSD		
Psychotic symptoms	3.1 (1.2)	
Disorganized thinking	2.5 (1.5)	
Negative symptoms	3.0 (1.1)	
Excitement/Mania	1.3 (1.3)	
Depression/Anxiety	2.1 (1.3)	
Total	12.0 (4.0)	

Abbreviations: BE-PSD, Brief Evaluation of Psychosis Symptom Domains; GASS-C-J, Japanese Version of Glasgow Antipsychotic Side-effects Scale for Clozapine

*From the Japan Association of Clinical Laboratory Standards, the Japan Diabetes Society.

### Reliability of the GASS-C-J

The Cronbach’s alpha coefficient of the GASS-C-J at the first and second rating was 0.78 (95% CI: 0.72 to 0.84) and 0.82 (95% CI: 0.76 to 0.88), respectively. The test-retest reliability of the total GASS-C-J score was evaluated by calculating the weighted kappa coefficient of individual and total GASS-C-J item scores ranged from 0.45 to 0.88 (Table 2).

**Table 2:** Test-retest reliability of the GASS-C-J.

GASS-C-J		Cohen's Kappa	p-value
1	I felt sleepy during the day	0.73	<0.01
2	I felt drugged or like a zombie	0.45	<0.01
3	I felt dizzy when I stood up or have fainted	0.61	<0.01
4	I have felt my heart beating irregularly or unusually fast	0.57	<0.01
5	I have experienced jerking limbs or muscles	0.69	<0.01
6	I have been drooling	0.69	<0.01
7	My vision has been blurry	0.46	<0.01
8	My mouth has been dry	0.60	<0.01
9	I have felt sick (nauseous) or have vomited	0.45	<0.01
10	I have felt gastric reflux or heartburn	0.52	<0.01
11	I have had problems opening my bowels (constipation)	0.58	<0.01
12	I have wet the bed	0.69	<0.01
13	I have been passing urine more often	0.47	<0.01
14	I have been thirsty	0.51	<0.01
15	I have felt more hungry than usual or have gained weight	0.62	<0.01
16	I have been having sexual problems	0.88	<0.01
Total		0.45	<0.01

Abbreviations: GASS-C-J, Japanese Version of Glasgow Antipsychotic Side-effects Scale for Clozapine

### Relationship between symptom severity of the BE-PSD and test-retest reliability of the GASS-C-J

The correlations between the test-retest discrepancy in each GASS-C-J item score and each BE-PSD item score are shown in Table 3. The following correlations were significant: psychotic symptoms and nausea/vomiting (r_s_ = 0.27), thirst (r_s_ = 0.31), and appetite/weight gain (r_s_ = 0.27); and disorganized thinking and urinary incontinence (r_s_ = 0.26); depression/anxiety and myoclonus (r_s_ = 0.25), hypersalivation (r_s_ = -0.27), and blurry vision (r_s_ = -0.22).

**Table 3:** Correlations between symptom severity of the BE-PSD and discrepancy in each item score of the GASS-C-J.

	BE-PSD									
	Psychotic symptoms		Disorganized thinking		Negative symptoms		Excitement/Mania		Depression/Anxiety	
	r_s_	p-value	r_s_	p-value	r_s_	p-value	r_s_	p-value	r_s_	p-value
**GASS-C-J**										
1	0.03	0.78	-0.1	0.36	-0.1	0.36	0.07	0.52	0.00	0.98
2	0.09	0.41	-0.05	0.64	0.01	0.92	0.15	0.17	0.13	0.23
3	-0.07	0.52	-0.18	0.11	0.03	0.77	-0.14	0.22	0.13	0.26
4	0.01	0.90	-0.10	0.36	-0.15	0.18	0.05	0.66	0.15	0.18
5	0.06	0.59	0.15	0.19	0.14	0.20	0.08	0.50	**0.25**	0.02
6	-0.04	0.71	-0.02	0.83	-0.05	0.66	0.07	0.51	**-0.27**	0.01
7	0.08	0.47	-0.04	0.75	0.03	0.76	-0.01	0.96	**-0.22**	0.05
8	0.20	0.07	-0.05	0.67	-0.13	0.23	0.13	0.23	0.09	0.42
9	**0.27**	0.02	0.09	0.42	0.14	0.20	0.13	0.24	0.12	0.27
10	-0.02	0.88	-0.10	0.35	0.05	0.68	-0.06	0.57	0.07	0.55
11	-0.05	0.67	0.04	0.72	0.07	0.54	-0.1	0.39	-0.06	0.57
12	0.13	0.25	**0.26**	0.02	0.05	0.62	0.14	0.21	0.05	0.65
13	0.18	0.11	-0.09	0.43	0.04	0.75	-0.02	0.84	-0.07	0.53
14	**0.31**	0.00	0.05	0.69	0.16	0.16	0.15	0.16	0.05	0.64
15	**0.27**	0.01	0.07	0.55	0.08	0.45	0.06	0.61	0.02	0.87
16	0.02	0.83	0.14	0.21	0.14	0.21	-0.04	0.72	-0.06	0.58

Abbreviations: BE-PSD, Brief Evaluation of Psychosis Symptom Domains; GASS-C-J, Japanese Version of Glasgow Antipsychotic Side-effects Scale for Clozapine

Bold number indicates p<0.05.

## Discussion

We developed the GASS-C-J by forward and backward translation, and assessed its reliability with more than 109 patients receiving clozapine treatment. To our knowledge, the GASS-C-J is the first self-administered scale available in Japanese to assess clozapine-related side effects. With regard to internal consistency, the Cronbach’α coefficient was approximately 0.8 for both the first and second evaluations. Although there is no clear consensus regarding the interpretation of the α coefficient, a value of ≥0.8 is generally regarded as high [22]. The weighted kappa coefficient for test-retest reliability also indicated “moderate agreement” to “almost perfect” agreement for all items in this study, in light of the criteria proposed by Landis and Koch [23]. These findings indicate that the GASS-C-J is a reliable scale to assess side effects associated with clozapine.

It should be noted that the reliability of patient-reported outcomes evaluated by self-rating scales including the GASS-C-J could be influenced by symptoms of schizophrenia [24]. For example, Takeuchi et al. reported that disorganization and cognitive dysfunction had an impact on the discrepancies in the scores of the Subjective Well-being under Neuroleptics Scale–Short form in patients with schizophrenia [24]. We also found that discrepancies in some GASS-C-J items between the two ratings were correlated with some symptom domains. In particular, discrepancies in nausea/vomiting (Q9), thirst (Q14), appetite/weight gain (Q15) showed positive correlations with psychotic symptoms, and discrepancy in myoclonus (Q5) was associated with depressive/anxiety symptoms. On the other hand, disorganized thinking was not associated with discrepancies in any of the GASS-C-J items except for incontinence (Q12). These findings suggest that self-ratings of subjective side effects (such as nausea) become more variable with a greater degree of psychotic symptoms, while fluctuation in neurological side effects (such as myoclonus) are predicted by depression/anxiety symptoms.

There are several limitations to be noted in the present study. First, the sample size was slightly smaller than that recommended by the COnsensus-based Standards for the selection of health Measurement INstruments (COSMIN) standard [25] to evaluate test-retest reliability of 16 items of the GASS-C-J, which is 112 (7 times the number of items). Second, we did not include any other scales to assess clozapine-related side effects and therefore were not able to evaluate the concurrent validity of the GASS-C-J. Third, the sensitivity to change of the GASS-C-J was not examined in this study. Fourth, the correlation analysis between discrepancies in the GASS-C-J item scores and psychopathology as assessed by the BE-PSD was not hypothesis driven, and we did not control for multiple analyses. Lastly, because the current study only included patients receiving a stable dose of clozapine, the reliability of the GASS-C-J cannot be guaranteed for patients during the early stage of clozapine treatment, in which patients more frequently experience side effects [26]. On the other hand, since the present study included both inpatients and outpatients, the findings are likely applicable to a wide range of patients taking clozapine.

In conclusion, the GASS-C-J demonstrated high internal consistency and good test-retest reliability, indicating the potential to be widely used in both clinical and research settings. The use of the GASS-C-J will enable physicians to elicit clozapine-associated side effects both efficiently and comprehensively, which is expected to improve timely management of these side effects and enhance adherence to clozapine treatment.
